# New constraints on axion-like dark matter using a Floquet quantum detector

**DOI:** 10.1126/sciadv.abl8919

**Published:** 2022-02-04

**Authors:** Itay M. Bloch, Gil Ronen, Roy Shaham, Ori Katz, Tomer Volansky, Or Katz

**Affiliations:** 1 School of Physics and Astronomy, Tel-Aviv University, Tel-Aviv 69978, Israel.; 2 Rafael Ltd., IL-31021 Haifa, Israel.; 3 Department of Applied Physics, Hebrew University of Jerusalem, 9190401 Jerusalem, Israel.; 4 Department of Physics of Complex Systems, Weizmann Institute of Science, Rehovot 76100, Israel.

## Abstract

Dark matter is one of the greatest mysteries in physics. It interacts via gravity and composes most of our universe, but its elementary composition is unknown. We search for nongravitational interactions of axion-like dark matter with atomic spins using a precision quantum detector. The detector is composed of spin-polarized xenon gas that can coherently interact with a background dark matter field as it traverses through the galactic dark matter halo. Conducting a 5-month-long search, we report on the first results of the Noble and Alkali Spin Detectors for Ultralight Coherent darK matter (NASDUCK) collaboration. We limit ALP-neutron interactions in the mass range of 4 × 10^−15^ to 4 × 10^−12^ eV/*c*^2^ and improve upon previous terrestrial bounds by up to 1000-fold for masses above 4 × 10^−13^ eV/*c*^2^. We also set bounds on pseudoscalar dark matter models with quadratic coupling.

## Teaser

Bounds are set on ultralight dark matter and its interaction with neutrons using a Floquet NMR quantum detector.

## INTRODUCTION

A plethora of multiscale astrophysical and cosmological evidence suggests that roughly 85% of the matter in our universe is unlike the matter we see around us. Despite almost a century of research, the evidence for this so-called dark matter (DM) is purely gravitational. As a result, its entire particle identity including its mass, spin, and interactions with itself and with other particles remains unknown.

Various theoretical models propose an abundance of candidates that would explain the physical nature of DM. A well-motivated one is a postulated particle named the axion (*1*, *2*), originally introduced to solve the strong CP problem (*3*). Over the years, many generalizations to the axion have been postulated, and they are collectively known as axion-like particles (ALPs) (*4*). These ALPs can be produced in the early universe and can account for the observed phenomena associated with DM. While the uncertainty for the ALP mass spans many orders of magnitudes, a particularly interesting range is that of ultralight masses (*5*). In this regime, the ALP De-Broglie wavelength is considerably longer than the length of the detector, and in addition, the number of particles within a single De-Broglie wavelength cubed (roughly the classical volume a single particle occupies) is much larger than 1. This implies that any interaction of ultralight ALPs with other particles such as protons, neutrons, electrons, and photons would be coherently enhanced and more easily detected (*6*–*14*). Moreover, the coupling between ultralight ALP DM with electron and nuclear spins can be manifested in the form of anomalous magnetic fields that induce an oscillatory energy shift at a characteristic frequency that depends on the ALP mass (*15*).

Various groups search for cosmological DM using astronomical observations and terrestrial detectors. Comagnetometers and nuclear magnetic resonance (NMR) sensors, in particular, are compact sensors that feature enhanced sensitivity to regular and anomalous magnetic fields. These sensors are composed of a dense ensemble of spin-polarized nuclei in a gaseous, liquid, or solid phase, whose collective response to regular or anomalous fields is measured by a precision magnetometer (*6*, *9*–*13*, *16*–*25*).

While these sensors have long been applied in various disciplines including medicine (*26*), chemistry (*27*, *28*), geology (*29*), physics, and engineering (*30*), their application for the search of cosmological DM is only at its infancy, with potential unprecedented sensitivities (*31*–*33*). Recently, these sensors have set new terrestrial constraints on the coupling of neutrons to ALP DM with masses *m*_DM_ ≲ 4 × 10^−13^ eV/*c*^2^ (*c*, speed of light) (*6*) by using an in situ atomic magnetometer. However, this in situ magnetometery is typically limited to measurements of small ALP masses, the reason for which can be traced back to the large difference between the gyromagnetic ratios of the nuclear spins and the electronic spins that comprise the two magnetometers.

Application of strong time-modulated fields, and Floquet engineering methods in particular, provides exquisite control over properties of materials. These methods have been widely applied in various disciplines in condensed matter and atomic physics, enabling control over the topology and band structure of materials (*34*, *35*), the formation of time crystals (*36*), and modification of the effective gyromagnetic ratio of atoms and their response to external fields (*37*, *38*). Utilization of Floquet fields have long enabled to enhance the sensitivity of NMR sensors at high frequencies (*39*), and recently, it was proposed as an eminent avenue to enhance the performance of DM field detectors (*40*). However, constraints on the coupling of DM (and in particular ALPs) to fermions using Floquet techniques have never been realized until this work.

**Fig. 1.**
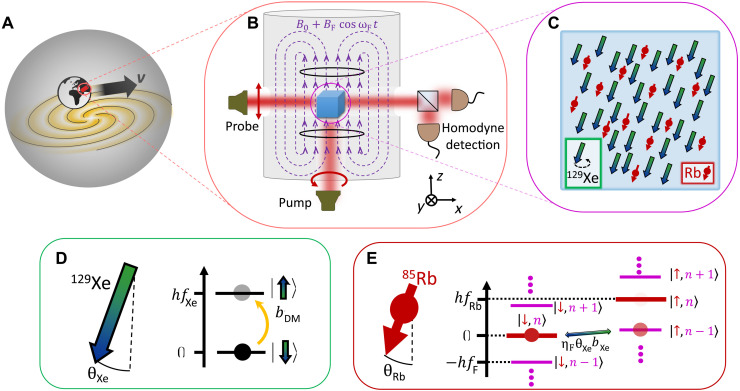
Floquet quantum detector for the search of ultralight axion-like DM. (**A**) As Earth moves across the Milky Way galaxy, it traverses the DM halo with a mean virial velocity *v*_vir_. (**B** and **C**) The Floquet detector is composed of a dense ensemble of spin-polarized ^129^Xe gas, which can resonantly interact with the moving axion-like DM. The interaction is in the form of an anomalous magnetic field, penetrating the detector shields that deflect regular magnetic fields. The spin precession is monitored via an in situ optical magnetometer using ^85^Rb vapor that is magnetically driven by a strong Floquet field *B*_F_. (**D**) Energy-level structure of the nuclear spin of ^129^Xe. The DM field oscillating near the NMR resonance frequency of the xenon with amplitude *b*_DM_ can drive collective spin flips of the ensemble in a coherent manner, rotating the net direction of the spin-polarized ensemble at an angle θ_Xe_. (**E**) Floquet spectrum of the ^85^Rb spins dressed by *n* RF photons. Collective spin flips of the polarized ^85^Rb ensemble by the slowly precessing xenon field (θ_Xe_*b*_Xe_) are greatly enhanced when the energy splitting of the Rb is large (*f*_Rb_ ≳ Γ_Rb_). For example, absorption of an RF photon of the Floquet field in the transition (∣↓, *n*⟩ → ∣↑, *n* − 1⟩) is enhanced by a factor η_F_ compared to a spin flip (∣↓⟩ → ∣↑⟩) in the absence of the Floquet drive (*n* = 0). This transition bridges between the large frequency mismatch of the electron (^85^Rb) and nuclear (^129^Xe) spin resonances and enables efficient detection at frequencies higher than previously measured.

Here, we report on new experimental constraints on the ALP-DM interactions with neutrons. The results, first from the NASDUCK collaboration (Noble and Alkali Spin Detectors for Ultralight Coherent darK matter), rely on measurements that took place over a period of 5 months using a dense spin-polarized ensemble of ^129^Xe atoms, whose response to anomalous fields was measured using an in situ precision rubidium Floquet magnetometer (see Fig. 1). The presence of the Floquet field enabled us to expand our search by more than an order of magnitude in masses, placing strong constraints in the mass range 4 × 10^−15^ eV/*c*^2^ < *m*_DM_ < 4 × 10^−12^ eV/*c*^2^. We improve on the current terrestrial limits on the coupling to neutrons by as much as three orders of magnitude. We also cast bounds on quadratic interactions (*13*), improving all existing bounds for some masses within the range of 2 × 10^−14^ eV/*c*^2^ < *m*_DM_ < 7 × 10^−13^ eV/*c*^2^ for neutron-DM quadratic interactions. Last, we also cast additional model-dependent bounds on the coupling of ALPs to protons and discuss their model uncertainty.

## RESULTS

### Interaction of ALP DM with spins

ALPs are pseudoscalars that can couple to neutrons in the form of an oscillatory magnetic-like field. The field amplitude *b*_DM_ is related to the DM local energy density ρ_DM_, whereas its frequency *f*_DM_ is related to the ALP DM mass *m*_DM_. The coupling of ALPs to spins is described by the interaction Hamiltonian

$$H=\gamma_{\text{Xe}}{\langle \text{cos}\;(2\pi f_{\text{DM}}t+\phi_{\text{DM}})b_{\text{DM}}\rangle }_{v,\phi_{\text{DM}}}\cdot I_{\text{Xe}}$$ (1)

where **I**_Xe_ denotes the nuclear spin-1/2 operator of xenon and γ_Xe_ denotes its gyromagnetic ratio. The DM field oscillates at frequency *f*_DM_ = *m*_DM_(*c*^2^ + **v**^2^/2)/*h* (and with a random initial phase ϕ_DM_), with **v** being its velocity. The DM’s wave function is a narrow wave packet in the frequency domain with a center slightly above *m*_DM_*c*^2^/*h* and a spread (as well as the offset from *m*_DM_*c*^2^/*h*) that depends on the velocity distribution of the DM, which is moving in the galaxy with respect to Earth [see (*41*) for further details]. According to the standard halo model, the mean and the SD of **v** are both of order the virial velocity *v*_vir_ ≈ 220 km/s (*42*), and as a result, the field remains coherent for a considerably long time $h/(m_{\text{DM}}v_{\text{vir}}^{2})$, which corresponds to about 2 × 10^6^ oscillations. In Eq. 1, 〈·〉_**v**, ϕ_DM__ denotes the averaging over the velocity and phase distributions [see (*41*) for further details], while **b**_DM_ is the anomalous magnetic field that is defined by

$$b_{\text{DM}}=\epsilon_{N}g_{\text{aNN}}\sqrt{2\rho_{\text{DM}}\hbar c^{3}}v/\gamma_{\text{Xe}}$$ (2)

Here, *g*_aNN_ is the ALP-neutron coupling coefficient and ρ_DM_ = 0.4 GeV/(*c*^2^cm^3^) (*42*). ϵ*_N_* is the fractional contribution of neutrons to the nuclear spin. Because the ^129^Xe nucleus has a valence neutron, the model uncertainty of ϵ*_N_* is relatively small, and contribution from coupling to its protons is about two orders of magnitude smaller. We adapt ϵ*_N_* = 0.63, corresponding to the smallest estimation in (*43*), using the ΔNNLO_GO_(394) model of the nuclear interactions. **b**_DM_ is considered an anomalous magnetic field because the Hamiltonian in Eq. 1 is independent of the gyromagnetic ratio (note that **b**_DM_ ∝ 1/γ_Xe_), which is associated with the spin coupling of fermions to regular magnetic fields and is thus unrelated to the interaction with ALPs.

In addition to ALP models, we also cast bounds on a pseudoscalar DM model, which interacts quadratically with neutrons (*13*). In this model, the anomalous field is given by

$$b_{\text{DM}}=\epsilon_{N}g_{N-\text{Quad}}^{2}\frac{2\rho_{\text{DM}}\hbar^{2}c^{2}}{m_{\text{DM}}}v/\gamma_{\text{Xe}}$$ (3)

Here, the DM oscillates at $f_{\text{DM}}^{\text{Quad}}(m_{\text{DM}})=f_{\text{DM}}^{\text{ALP}}(2m_{\text{DM}})$, implying that bounds on quadratic-type interactions are derived in the mass range 2 × 10^−15^ eV/*c*^2^ < *m*_DM_ < 2 × 10^−12^ eV/*c*^2^.

### Experimental setup and detection mechanism

The heart of our sensor consists of spin-polarized ^129^Xe atoms, whose collective response to time-varying fields is detected via an in situ precision optical magnetometer made of rubidium vapor. The atoms are encapsulated in a small cubical glass cell that is maintained at 150°C and surrounded by magnetic shields as shown in Fig. 1. We continuously polarize the Rb spins at their electronic ground state via optical pumping. The Rb polarizes, in turn, the nuclear spins of ^129^Xe via spin-exchange collisions along the $\hat{z}$ axis, effectively maintaining ∼3 × 10^16^ fully spin-polarized nuclei. Further details on the experimental configuration are described in Materials and Methods and (*41*).

An anomalous DM field pointing in the *xy* plane of the detector and interacting with the ^129^Xe nucleons would collectively tilt them off the $\hat{z}$ axis by an angle θ_Xe_ and force their precession at a frequency *f*_DM_. In the presence of an axial magnetic field, this tilt is suppressed outside of a narrow frequency band with width Γ_Xe_ centered around the NMR frequency *f*_Xe_ (corresponding to the precession frequency around the axial field) [We note that, in our setup, $f_{\text{Xe}}=\gamma_{\text{Xe}}(B_{\text{ext}}^{z}+b_{\text{Rb}})$, where $B_{\text{ext}}^{z}$ is the external field in the *z* direction and *b*_Rb_ is the effective magnetic field induced by the Rb atoms via the spin-exchange interactions]. Thus, for *f* = *f*_DM_

$$\theta_{\text{Xe}}(f=f_{\text{DM}})=\frac{\gamma_{\text{Xe}}\;b_{\text{DM}}}{2|i\Gamma_{\text{Xe}}+f_{\text{Xe}}-f_{\text{DM}}|}$$ (4)

Here, γ_Xe_ = −1.18 kHz/G is the gyromagnetic ratio of xenon, and Γ_Xe_ ≈ 0.3 Hz is the measured decoherence rate of the xenon spins. From Eq. 4, we thus learn that the noble-gas spins efficiently respond to the anomalous ALP field if it oscillates at a frequency *f*_DM_ that resonates with the NMR frequency *f*_Xe_. Our setup is capable of efficiently sensing *f*_DM_ in the 1- to 1000-Hz range.

To measure the precession of the noble-gas spins, we use the rubidium as an optical magnetometer. Using a linearly polarized optical probe beam, we measure the collective spin of the rubidium along the $\hat{x}$ axis via its imprint on the polarization of the probe beam, which rotates after traversing the alkali medium. The polarization rotation is subsequently measured with a set of differential photodiodes in a homodyne configuration (*44*–*46*). While the polarized rubidium spins are initially oriented along the $\hat{z}$ axis, they are tilted by an angle

$$\theta_{\text{Rb}}(f=f_{\text{DM}})=\frac{\gamma_{\text{Rb}}b_{\text{Xe}}\theta_{\text{Xe}}}{|i\Gamma_{\text{Rb}}+f_{\text{Rb}}-f_{\text{DM}}|}$$ (5)

Here, *b*_Xe_θ_Xe_ is the transverse spin-exchange field. *b*_Xe_ is proportional to the magnetic field produced by the spin-polarized xenon atoms and is enhanced by a large factor of κ_0_ = 518 owing to the Fermi contact interaction with the rubidium (*47*). We stress that this factor is a virtue of having an in situ magnetometer and allows for an improved sensitivity of the detector. *f*_Rb_ is the electron paramagnetic resonance (EPR) frequency (analogous to the NMR frequency for nuclear spins) of ^85^Rb spins, γ_Rb_ = 467 kHz/G is the gyromagnetic ratio of the ^85^Rb isotope (72% abundance), and Γ_Rb_ = 6.6 kHz is their decoherence rate.

The angle θ_Rb_ that is measured by the optical magnetometer is proportional to the DM anomalous field *b*_DM_. To detect this field efficiently, Eqs. 4 and 5 indicate that both the NMR and EPR frequencies should be brought in resonance with the anomalous DM field, satisfying ∣*f*_Xe_ − *f*_DM_∣ ≲ Γ_Xe_ and ∣*f*_Rb_ − *f*_DM_∣ ≲ *a*_Rb_ simultaneously. While both NMR and EPR frequencies depend linearly on the magnetic field $B_{\text{ext}}^{z}$ and have small nonzero offsets, their substantially different slopes γ_Xe_ and ∣γ_Rb_∣≈∣400γ_Xe_∣ hinder simultaneous resonance for high frequencies, where *f*_DM_ ≫ (γ_Xe_/γ_Rb_)Γ_Rb_. Thus, in this regime, while the NMR condition may be satisfied, the alkali spins remain out of resonance and the detector’s sensitivity is strongly impeded.

To address this problem and increase the range of masses in which DM can be detected, we apply an additional strong Floquet field that enhances the response of the detector and bridges the NMR and EPR frequency gap. The application of the Floquet field enables one to recover the sensor sensitivity at high axial fields, bridging the resonance mismatch between the bare magnetic resonances of the nuclear and alkali spins. Characterization of the Floquet operation and characterization of the detector’s background and performance are detailed in Materials and Methods.

### The search

#### Data acquisition

We search for anomalous magnetic fields oscillating in the range of *f*_DM_ = 1 to 1000 Hz (corresponding to *m*_DM_ in the range of 4 × 10^−15^ to 4 × 10^−12^ eV/*c*^2^). To maximize the realized sensitivity, for each searched frequency, we tuned the axial magnetic field to bring the NMR frequency $f_{\text{Xe}}(B_{\text{ext}}^{z})$ near resonance with *f*_DM_ and typically recorded the sensor response for (2 − 20) × 10^5^ oscillations. To cover all frequencies within the search range, we scanned the magnetic field in fine steps of about 0.2 mG to maintain overlap in the NMR frequencies of neighboring measurements. We have scanned the entire range of frequencies several times by slightly shifting the NMR frequencies to get ample measurements of any given frequency at different sensitivities. As a result, the search consisted of almost 3000 measurements, taken during a period of 5 months. To characterize the sensor response and validate its stability during the long search period, each measurement was preceded and followed by a set of calibration measurements. The calibration measurements automatically tuned the parameters of the oscillatory Floquet field and characterized the response function of the sensor. Data processing is described in Materials and Methods, and detailed search and calibration protocols are given in the Supplementary Materials (*41*).

#### Detection capability

As DM ALP signals feature extremely long coherence time compared to the sensor backgrounds, they can potentially be directly detected and distinguished from the background. In the frequency domain, a signal centered at *f*_DM_ would have an ultranarrow bandwidth with a quality factor of about ∼2 × 10^6^, thus being distinguishable from the noise that is approximately white within that bandwidth [see (*41*)]. Sideband analysis further enables to differentiate between the signal and background. We use measurements at magnetic fields in which the NMR frequency is off-resonant and the sensitivity to anomalous fields is negligible as control measurements. These measurements enable us to identify frequencies in which the background has coherent properties that require specialized analysis procedures that rely on the different responses instead of the coherence times of signal and background. Last, multiple repetitions of measurements enable to exclude transient noise and differentiate it from the coherent ALP signal.

### Search results

**Fig. 2.**
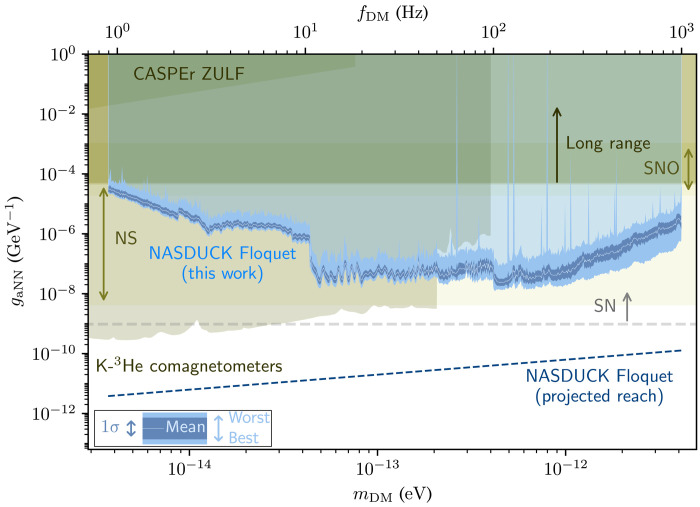
Constraints on ALP-neutron couplings. In this work, we derive the constraints using the Floquet quantum detector by the NASDUCK collaboration as a function of the ALP mass. All presented constraints calculated in this paper correspond to 95% C.L. of the bound. Because of the finite resolution of the figure and given the dense set of measurements, the limits appear as a bright blue band. The width of this band denotes the strongest and weakest values around each mass point. The precise tabulated bounds can be found in (*48*). The bright blue solid line shows a binned average of the bound, while its 1σ variation is shown in dark blue band (both calculated in log space at a binning resolution of 1% of the mass). The light transparent blue region shows the exclusion region for the ALP-neutron couplings. The dashed dark blue line shows the projected sensitivity of this experiment, as discussed in the text. The olive green regions show other terrestrial constraints, including the CASPEr ZULF experiments (*12*, *49*), K-^3^He comagnetometer bounds (*6*, *18*, *50*), and long-range constraints on ALP-neutron (*18*) couplings. In beige, the agreed astrophysical excluded region from solar ALPs unobserved in the SNO (*51*) and from NS cooling (*52*, *66*–*68*) is shown. The region above the gray dashed line is excluded by SN cooling considerations and neutrino flux measurements (*4*, *53*, *54*). The SN cooling constraint strongly relies on the unknown collapse mechanism, and hence, the limits should be taken with a grain of salt (*55*).

We use the log-likelihood ratio test to constrain the presence of ALP DM with 95% confidence level (C.L.) bounds, presented on ALP-neutron couplings in Fig. 2. The constraints cover the entire mass range between 4 × 10^−15^ and 4 × 10^−12^ eV/*c*^2^, measured with high resolution roughly set by the reciprocal measurement time. The resulting limits are presented by the bright blue band and are tabulated in (*48*). Because of the finite resolution of the figures, the limits appear simply as a bright blue band whose width reflects the strongest and weakest values around each frequency point. To convey the typical bound, we further present a binned average of the bound (bright blue solid line) and its 1σ variation (dark blue band), both calculated in log space at a binning resolution of 1% of the mass. Notably, all values of the ALP-neutron couplings above the bright blue band are excluded (light transparent blue region). The olive green regions show other terrestrial constraints, including the CASPEr ZULF experiments (*12*, *49*), K-^3^He comagnetometer bounds (*6*, *18*, *50*), and long-range constraints on ALP-neutron (*18*) couplings. In beige, the astrophysical limits from not observing solar ALPs in the Solar Neutrino Observatory (SNO) (*51*) and neutron star (NS) cooling considerations from ALP-neutron interactions (*52*) are shown. The region above the dashed gray line is excluded by supernova (SN) cooling considerations and neutrino flux measurements (*4*, *53*, *54*).

**Fig. 3.**
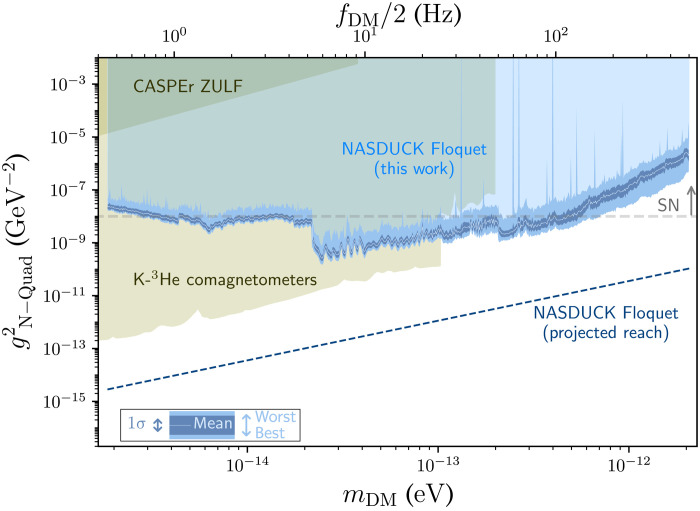
Constraints on neutron-DM couplings of quadratic type. The quadratic constraints (*13*) are derived as a function of the ALP mass, using the Floquet quantum detector by the NASDUCK collaboration in this work. All presented constraints calculated in this paper correspond to 95% C.L. of the bound. As in Fig. 2, because of the finite resolution of the figure and given the dense set of measurements, the limits appear as a bright blue band. The width of this band denotes the strongest and weakest values around each mass point. The precise tabulated bounds can be found in (*48*). The bright blue solid line shows a binned average of the bound, while its 1σ variation is shown in dark blue band (both calculated in log space at a binning resolution of 1% of the mass). The light transparent blue region shows the exclusion region for this model. The dashed dark blue line shows the projected sensitivity of this experiment, as discussed in the text. The olive green regions show other terrestrial constraints, including the CASPEr ZULF experiments (*12*, *49*) and the recasted K-^3^He comagnetometer bounds (*6*, *18*, *50*). The region above the dashed gray line is excluded by SN cooling considerations (*13*). We stress that the SN cooling constraint strongly relies on the unknown collapse mechanism, and hence, the limits should be taken with a grain of salt (*55*).

Figure 3 presents with constraints on neutron-DM couplings of quadratic type. The new bounds of the NASDUCK Floquet detector use the same coloring conventions as the bounds of the ALP-neutron interactions. Similarly, the olive green regions show other terrestrial constraints, from the CASPEr ZULF experiment (*12*, *49*), and bounds converted in (*6*), using data from (*18*, *50*) from K-^3^He comagnetometers. The regions above the dashed gray lines are excluded by the more uncertain [see (*55*) for further details] bounds that arise from SN cooling considerations (*13*).

Current models of the nuclear structure of ^129^Xe predict that its spin composition has a nonzero fractional contribution from protons ϵ*_P_*. If ϵ*_P_* is nonzero, then the search data can also be used to cast additional bounds on the coupling strength of ALPs with protons by carrying the substitution ϵ*_N_g*_aNN_ → ϵ*_P_g*_aPP_ in Eq. 2 (assuming that *g*_aNN_ = 0 for this particular likelihood test). In (*41*), we present the bounds obtained by our data for four different values of ϵ*_P_* associated with different nuclear structure models (*43*, *56*). For these values, the new model-dependent bounds we derive improve other terrestrial bounds on the coupling of ALPs with protons. However, we strictly emphasize that the reliability of these model-dependent proton coupling bounds should be taken with a grain of salt. The uncertainties of the nuclear structure models could be quite large as they are not sufficiently quantified for ^129^Xe (*57*), and possibly ϵ*_P_* could even vanish.

Estimation of future performance for casting stronger bounds than our current detector is presented by the dashed dark blue line based on usage of low-noise ferrite shields (*58*) and multiple passages of the probe beam within the cells (*59*), expecting a white noise floor of $1\;\text{fT}/\sqrt{\text{Hz}}$. The reach is estimated for measurements of 2 × 10^6^ oscillations for each frequency, corresponding to about 2 years of measurement using a single detector.

Our newly derived limits on the ALP-neutron coupling substantially improve the existing terrestrial limits in the mass range of 2 × 10^−13^ to 4 × 10^−12^ eV/*c*^2^, complementing the yet stronger astrophysical constraints. Notably, astrophysical constraints typically suffer from substantial systematic uncertainties that render them less certain.

## DISCUSSION

In summary, we presented new constraints on the ALP DM couplings to neutrons, substantially improving previous bounds. Our detector used dense xenon spins and in situ Floquet magnetometer, which enabled the extension of ALP masses to higher values. In addition, new bounds on neutron-quadratic type interactions were also cast.

NMR detectors are frontier technology to search for new physics, which, for ALPs, can potentially reach the limits of QCD axions in the standard quantum limit (*33*). Practically, however, these sensors are often limited by the magnetic noise floor and the realized detector sensitivity that nonetheless are expected to exceed astrophysical bounds for coupling with neutron spins. In addition, similar searches using nuclei whose proton spin component is known with higher certainty could extend such searches and cast reliable bounds on the coupling of ALPs with protons.

### Note added

Toward the end of our search and during the late stages of the analysis of the recorded data, we became aware of the study of Jiang *et al*. (*60*), which uses an NMR detector to search for nucleon-ALP coupling. Jiang *et al*.’s (*60*) study finds similar sensitivity, but it does not use Floquet, it does not account for ϵ*_N_*, and it does not account for the stochastic effects of the ALPs. In addition, Gramolin *et al*. (*61*) have recently independently shown how to account for the stochastic properties of the ALPs, in a method that is similar to our detailed analysis in (*41*).

## MATERIALS AND METHODS

### The Floquet bandwidth enhancement

The Floquet field $B_{F}\text{cos}\;(2\pi f_{F}t)\hat{z}$ is aligned with the previously described axial field, $B_{\text{ext}}^{z}$. The strong field modulates the ground-state energy of the rubidium spins, thereby dressing their energy levels with the field induced by the radio frequency (RF) photons (*62*). This modulation is spectrally manifested as a series of resonance bands that appear at discrete harmonics of the driving field in the magnetic spectrum of the rubidium, and via multiphoton processes, it encodes the response of the spins to low-frequency fields. The resulting tilt of the measured rubidium is thus obtained from Eq. 5 by shifting the spectrum by *nf*_F_ with an integer *n* and multiplying the response with a bandwidth enhancement factor $\eta_{F}^{\left(n\right)}$ such that around the harmonics of the Floquet frequency $\theta_{\text{Rb}}^{\text{Floquet}}(f=f_{\text{DM}}+nf_{F})=\eta_{F}^{\left(n\right)}\theta_{\text{Rb}}(f=f_{\text{DM}})$.

**Fig. 4.**
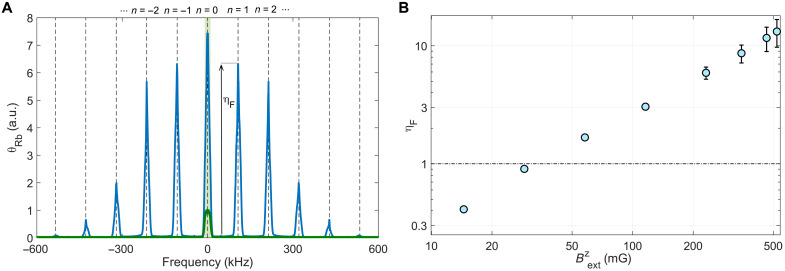
The Floquet modulation and the corresponding effective enhancement of the bandwidth. (**A**) The response (root mean square) in arbitrary units (a.u.) of the optical (Rb) magnetometer (green line) to an input low-frequency transverse magnetic noise in the absence of the Floquet field. The injected noise spectral content is cut off at 10 kHz (light green shade), and the axial magnetic field is taken to be $B_{\text{ext}}^{z}=0.23G$, corresponding to a resonance frequency of *f*_Rb_ = 105 kHz. In the absence of the Floquet modulation, the response has the same spectral support as the input field and its peak is normalized to unity. In the presence of a strong axial Floquet field, the magnetometer response (blue line) is altered substantially, mapping and enhancing the input field at integer multiples of the Floquet frequency (indicated with dashed lines). η_F_ denotes the enhancement of the magnetic response near the first Floquet band over the maximal response in the absence of the Floquet field. (**B**) Measured enhancement bandwidth factor, η*_F_*, as a function of the axial magnetic field. The Flouqet modulation, which is tuned to the Rb resonance frequency, is shown to improve the magnetometer response over the unmodulated case and by that enable recovering of much of its maximal sensitivity (dashed line).

In Fig. 4A, we exemplify the Floquet spectrum via typical measurement of the spectrum, θ_Rb_(*f*), in response to a low-frequency transverse magnetic noise, whose variance is white up to a cutoff frequency set at *f*_c_ = 10 kHz (light green shade), while setting *f*_Rb_ = 105 kHz. In the absence of the Floquet field, the rubidium response appears at the same frequencies of the drive (green line), and the response is suppressed because the driving field is tuned away from resonance ∣*f*_Rb_ − *f*_c_ ∣ ≃ 14Γ_Rb_. By applying a strong and resonant Floquet field (*f*_F_ = *f*_Rb_, *B*_F_ = 0.4 G), the low-frequency spectrum is mapped to discrete harmonics of the Floquet field at integer multiples *n* for which efficient coupling is realized. We observe an $\eta_{F}^{\left(1\right)}=5.9$-fold enhancement of the magnetic response near the first Floquet band with respect to the low-frequency response of the unmodulated sensor. Notably, routine calibrations of the Floquet parameters during the search did not inject magnetic noise but used coherent sinusoidal signals, following the protocol that is described in detail in (*41*).

The resonant nature of the Floquet modulation is encoded in $\eta_{F}^{\left(n\right)}$, which depends on the external magnetic field and the Floquet amplitude, *B*_F_. In Fig. 4B, we show the enhancement, $\eta_{F}\equiv \eta_{F}^{\left(1\right)}$, as a function of the axial magnetic field. At each measured value, we optimized for the frequency and amplitude of the Floquet field, following the theoretical analysis detailed in (*41*).

### Background and signal sensitivity

The sensor is susceptible not only to the anomalous fields but also to various sources of noise that limit its detection sensitivity. In this section, we present and characterize the noise model for the detector, whereas the protocol that routinely monitored and calibrated for variations of the model parameters during the search is described in (*41*). The dominant sources of noise are the magnetic field noise, δ*B*, and optical polarization noise due to the shot noise of the probe beam *W*. In the presence of the former, Eqs. 4 and 5 are modified by taking *b*_DM_ → *b*_DM_ + δ*B* and *b*_Xe_θ_Xe_ → *b*_Xe_θ_Xe_ + δ*B*. To study the response of the system to the above noise, we combine Eqs. 4 and 5 and rewrite the Floquet-demodulated output of the optical magnetometer *S* + *N*, decomposed to the noise contribution

$$N(f)=\xi \frac{\delta B(f)}{1+i(f_{\text{Xe}}-f)/\Gamma_{\text{Xe}}}+\delta B(f)+W(f)$$ (6)

and the coherent signal contribution of the ALP at *f* = *f*_DM_

$$S(f)=\xi \frac{b_{\text{DM}}}{1+i(f_{\text{Xe}}-f)/\Gamma_{\text{Xe}}}$$ (7)

The detector reading, *S* + *N*, is measured around the first Floquet band and is given here in units of magnetic field. The calibration protocol of the magnetometer, which determines the proportionality constant that converts the measured optical signal to magnetic field units, is described in section S3 in (*41*). Here, *W* denotes the optical probe noise obtained after demodulation. ξ = γ_Xe_*b*_Xe_/2Γ_Xe_ is the overall dimensionless factor that encodes the enhancement of the rubidium response to the xenon precession over the direct impact of magnetic noise. This is in line with the view of the xenon precession as the signal (also affected by regular and anomalous magnetic fields) measured via the optical magnetometer. ξ is calibrated routinely, and during the entire search, its value ranged from 1 to 3 [see (*41*) for further details]. For ξ ≫ 1 and ξ∣δ*B* ∣≫∣*W*∣, the experimental setup reaches a maximal sensitivity to the ALP couplings (*g*_aNN_) and is limited solely by the magnetic field noise and the gyromagnetic ratio of the nuclei. In the absence of a noise-cancellation mechanism, this limit is universal to all types of NMR sensors, independent of the number of polarized nuclear spins, the performance of the used magnetometer, or the coherence times of the atoms.

**Fig. 5.**
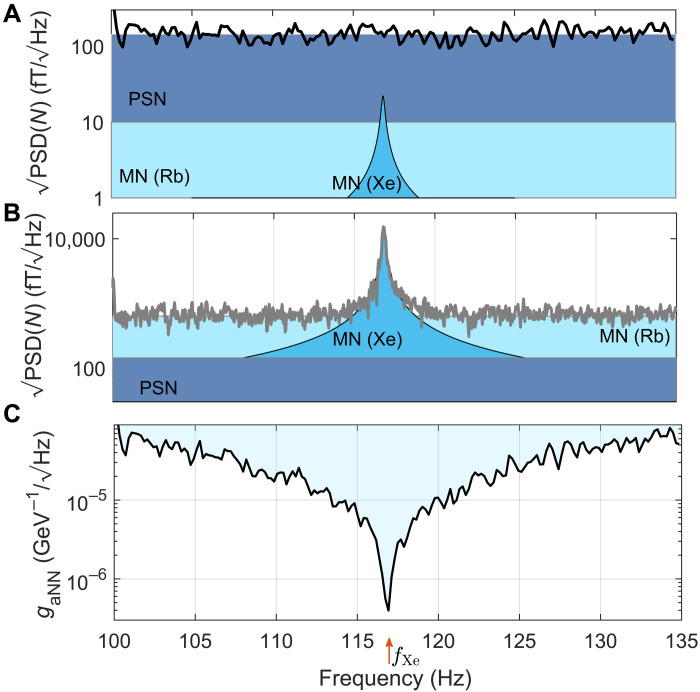
Noise spectral density measurement and its implication on the detector sensitivity. (**A**) Measured noise spectral density PSD(*N*) (black line) near the xenon resonance frequency in the presence of a Floquet field and an axial field $B_{\text{ext}}^{z}=0.1G$. The shaded regions denote the different estimated noise contributions of optical photon shot noise (PSN; dark blue) and magnetic noise (MN) coupled to Rb spins (light blue) and Xe spins (blue). (**B**) Sensor response (gray line) to an injected, white magnetic noise $\delta B_{x}=5.6\text{pT}/\sqrt{\text{Hz}}$. By tuning the magnetometer parameters to be sensitive predominantly to one direction in the *xy* plane, one is able to disentangle the response of the xenon spins from the injected noise. The figure shows the resonant behavior of the xenon in the presence of a dominant magnetic noise. Shaded regions are the same as the top figure. (**C**) Sensitivity to anomalous fields as inferred from the calibrated response of the magnetometer and the noise characteristics in (A), assuming coherent and deterministic ALPs (black line). When the photon shot noise (flat) spectrum is dominant, the signal and hence the sensitivity are enhanced near the xenon resonance frequency (red arrow).

To exemplify the noise characteristics of the detector, in Fig. 5, we present the square root of the spectral density of a noise measurement for a single recording at $B_{\text{ext}}^{z}=0.1\;G$. In this measurement, no coherent calibration signals (other than the Floquet drive) are present, and the spectral density of the recorded noise realization PSD(*N*) is calculated using Welch’s method. The noise spectrum has a typical square root of spectral density of $100\;\text{fT}/\sqrt{\text{Hz}}$ (dark blue shaded region) and is dominated by noise of the probe beam *W*, which we show in (*41*) to be governed by photon shot noise. The estimated contribution of the magnetic noise is ≈10 fT/tHz, generated by the inner layer of the magnetic shield (*63*) and sensed by the rubidium (light blue region) and xenon (blue region) spins. We find that only at a few frequencies (and, in particular, at harmonics of the mains hum) the magnetic noise becomes dominant over the probe noise.

To characterize the response to transverse fields, we inject white magnetic noise along the $\hat{x}$ direction with a spectral density of $5.6\;\text{pT}/\sqrt{\text{Hz}}$ as presented in Fig. 5B. To highlight the response of the xenon nuclei, we tune the Floquet parameters of the rubidium vector magnetometer to be most sensitive to fields along the $\hat{y}$ direction, suppressing the response to $\hat{x}$ fields. Thus, we gain sensitivity to the response of the xenon spins to the injected noise while suppressing its direct effect on the rubidium magnetometer. The Lorentzian response of the xenon near its NMR frequency validates Eq. 7 and enables also to estimate its parameters, yielding ξ = 2.2. The injection of magnetic noise to characterize the detector was used only for the particular demonstration in Fig. 5B to highlight the coupling of magnetic noise and its decomposition to contributions of Rb and Xe. The routine calibration protocols that calibrate ξ use coherent sinusoidal signals instead as described in (*41*).

The detailed and strict search procedure and the methodology of the data analysis are described in (*41*), taking into account the exact measurements of the search and the statistical properties of the ALPs. Nonetheless, we find it insightful to exemplify the spectral sensitivity to anomalous fields of the detector based on a short measurement of the sensor in the limit of *v*_vir_ → 0 as shown in Fig. 5C. It is apparent that, when white noise dominates the spectrum, the sensitivity follows the Lorentzian NMR shape of the nuclear spins, and a high sensitivity is limited to a narrow frequency band, whose width is determined by the xenon’s spectral width Γ_Xe_ = 0.3 Hz.

It is also possible to give an approximate estimate of the excluded *b*_DM_ and the typical θ_Xe_ from the measurement in Fig. 5A at *f*_DM_ ≈ 116 Hz in resonance with the NMR frequency. For the characteristic noise spectral density $\sqrt{\text{PSD}(N)}\approx 100\;\text{fT}/\sqrt{(}\text{Hz})$, a finite measurement interval *T* ≈ 3000 s, which is shorter than the ALP coherence time, and ξ = 2.2, we can exclude coherent anomalous fields that are larger than $b_{\text{DM}}≳\sqrt{\text{PSD}(N)}/(\xi \sqrt{T})\approx 1\;\text{fT}$. This field also corresponds to a minimal tilt of the Xe spins by about θ_Xe_ ≳ 2 × 10^−8^, obtained by Eq. 4 for Γ_Xe_ = 0.3 Hz. Note that these approximate estimates compared the power of the signal to the noise variance to determine exclusion [signal-to-noise ratio (SNR) = 1], whereas the actual constraints presented in Fig. 2 effectively set higher SNR to obtain the 95% C.L. in the likelihood test.

### Data processing

We automatically excluded data from our analysis if it matched at least one of the following criteria (quality cuts): (i) substantial variation in the sensor parameters between two subsequent calibration measurements, (ii) saturation of the detector that is identified via substantial decrease in the noise variance, and (iii) a substantial increase of transient noise, which is identified in spectral regions of the signal being far away from the NMR resonance. For (i), we veto the entire measurement, whereas for cuts related to both (ii) and (iii) data vetoing is limited to a finite measurement window.

We analyze the data using the log-likelihood ratio test to constrain the presence of ALPs at frequency *f*_DM_ with a width determined by the signal coherence time and the effects of Earth’s rotation on the sensitive axes of the detector. Each measurement was used to constrain a frequency range of width 2 to 6 Hz around the NMR frequency. Bounds were set on ALP-neutron interactions, as well as quadratic interactions (*13*) with neutrons. To accurately account for the velocity distribution of the DM (see Eq. 1), we followed the suggested analysis in (*19*). We used the asymptotic formulas in (*64*) for the distribution of the log likelihood. For each measurement, the noise was assumed to be white and was estimated using sideband analysis in the frequency domain away from the NMR resonance.

All analysis procedures and cuts were designed in a blinded fashion, and decided in advance before looking at the data, to eliminate bias. However, after unblinding, we found that less than 0.1% of the spectral domain of the search range was statistically inconsistent with the white noise model. For this part, we have refined the statistical tests to treat transient and coherent magnetic noise. All data are found consistent with the refined model. The ALP stochastic properties, the statistical analyses, and post-unblinding changes are detailed in (*41*).

## Supplementary Materials

### This PDF file includes:

Sections S1 to S8 Figs. S1 to S7 Table S1 References
